# Lactate in cardiogenic shock: pathophysiology, prognostic value, and clinical interpretation

**DOI:** 10.1016/j.aicoj.2026.100061

**Published:** 2026-04-20

**Authors:** Bruno Levy, Glenn Hernandez, Hamid Merdji

**Affiliations:** a Université de Lorraine, CHRU Nancy, Médecine Intensive et Réanimation, Nancy, France; b Université de Lorraine, INSERM U1116, Nancy, France; c Departamento de Medicina Intensiva, Facultad de Medicina, Pontificia Universidad Católica de Chile, Santiago, Chile; d Department of Medical Intensive Care, University Hospital of Strasbourg, Nouvel Hôpital Civil, Strasbourg, France; e INSERM (French National Institute of Health and Medical Research), UMR 1260, Regenerative Nanomedicine (RNM), FMTS, Strasbourg, France

**Keywords:** Lactate, Cardiogenic shock, Epinephrine, ECMO

## Abstract

In cardiogenic shock (CS), blood lactate concentration is routinely used to assess shock severity and guide clinical decisions. However, lactate interpretation is often oversimplified, leading to confusion between disease severity, shock reversibility, and treatment failure. A clinically grounded understanding of lactate behavior throughout the course of CS is therefore required. In CS, lactate elevation primarily reflects the magnitude and duration of systemic hypoperfusion caused by reduced cardiac output, often compounded by regional ischemia, particularly in the splanchnic territory. In addition to anaerobic mechanisms, lactate production is influenced by adrenergic stimulation, post–cardiac arrest syndrome, ischemia–reperfusion injury, systemic inflammation, and mitochondrial dysfunction. Impaired hepatic and renal clearance further contributes to sustained hyperlactatemia, explaining why lactate may remain elevated despite apparent restoration of macrocirculatory variables. Clinically, admission lactate is a robust marker of initial shock severity and is consistently associated with early mortality. However, once resuscitation has started, isolated lactate values provide limited information. Serial measurements and lactate trajectories over time more accurately reflect metabolic recovery and response to therapy. Early lactate clearance identifies patients with reversible shock physiology, whereas persistent or rising lactate levels indicate refractory shock, ongoing microcirculatory dysfunction, or impaired clearance. In CS patients requiring mechanical circulatory support, particularly veno-arterial extracorporeal membrane oxygenation (VA-ECMO), lactate plays a central role in clinical assessment. Pre-implantation lactate reflects disease severity but should not be interpreted as a stand-alone criterion for futility. Following VA-ECMO initiation, early lactate clearance is one of the strongest predictors of survival, while persistent hyperlactatemia despite adequate device flow is associated with multiorgan failure and poor outcome. In CS, lactate should be interpreted as an integrative and dynamic biomarker reflecting the balance between hypoperfusion, metabolic stress, and clearance rather than tissue hypoxia alone. Trajectory-based lactate assessment, closely aligned with clinical context and circulatory support strategies, provides critical information for risk stratification, therapeutic guidance, and evaluation of shock reversibility.

## Introduction

**Fig. 1:**
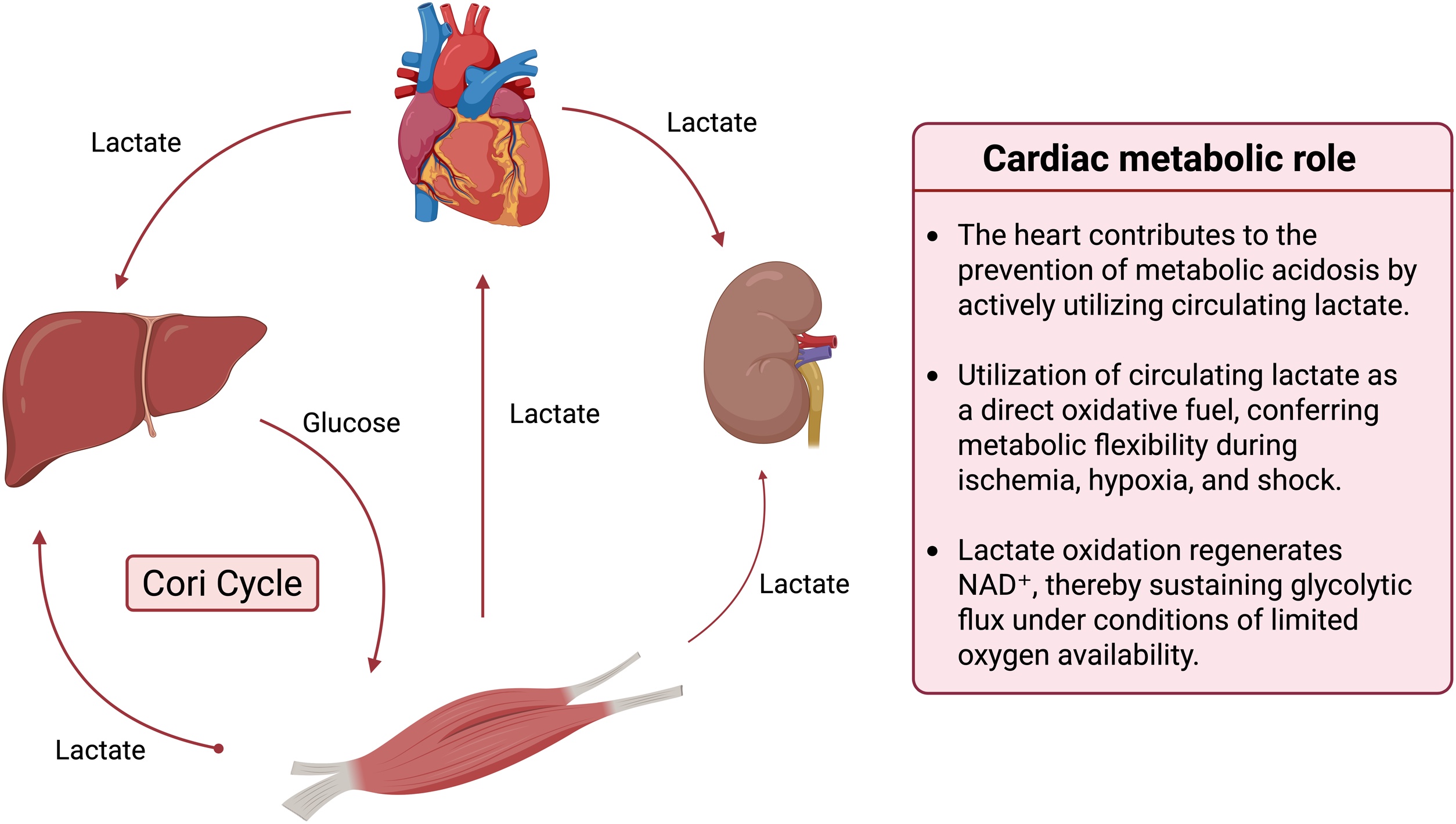
Role of the heart in systemic lactate metabolism and energy homeostasis (adapted from [83]). Circulating lactate is continuously exchanged between peripheral tissues, liver, kidneys, and the heart through coordinated metabolic pathways. Circulating lactate is readily taken up by the myocardium, where it serves as an important direct oxidative energy source, contributing to metabolic flexibility during ischemia or hypoxia. By supporting lactate utilization and regeneration of oxidized NAD^+^, cardiac lactate metabolism helps sustain glycolysis and may limit the development of metabolic acidosis. Under conditions of limited oxygen availability (e.g., intense exercise, ischemia, hypoxia, or shock), glucose predominantly in skeletal muscle is metabolized through anaerobic glycolysis, generating pyruvate that is subsequently reduced to lactate and released into the systemic circulation. Circulating lactate is taken up by the liver, where it is oxidized back to pyruvate and used as a substrate for gluconeogenesis, resulting in the regeneration of glucose. This newly synthesized glucose is then released into the bloodstream and reutilized by peripheral tissues, thereby completing the Cori cycle. While muscle gains 2 ATP per glucose during anaerobic glycolysis, hepatic glucose regeneration consumes 6 ATP equivalents, yielding a net whole-body cost of 4 ATP and highlighting the energetic inefficiency but metabolic indispensability of the Cori cycle. Excess lactate can also be cleared by the kidneys

Cardiogenic shock (CS) is the most severe presentation of acute heart failure and is characterized by a critical reduction in cardiac output leading to systemic hypoperfusion, cellular dysoxia, and progressive multi-organ dysfunction [1]. Despite advances in early revascularization, pharmacological therapy, and temporary mechanical circulatory support (t-MCS), mortality remains high, ranging from 30% to more than 50% in contemporary cohorts [[1], [2], [3], [4]], making it one of the most lethal forms of circulatory shock [5,6]. Among the numerous clinical, hemodynamic, and biological markers used to assess severity and guide management, blood lactate concentration has emerged as one of the most robust and reproducible prognostic indicators in CS [[7], [8], [9], [10], [11]]. Lactate measurement is part of the SHARC (Scientific Expert Panel From the Shock Academic Research Consortium) criteria for diagnosing CS, where a level >2 mmol/L serves as an optional marker of tissue hypoperfusion [12]. The revised Society for Cardiovascular Angiography and Interventions (SCAI) staging system for CS integrates new lactate thresholds with Stages A and B characterized by normal lactate concentrations, Stages C and D >2 mmol/L, and Stage E >8 mmol/L [13]. Furthermore, serial lactate monitoring is strongly endorsed by both the ACC Expert Consensus Statement Concise Clinical Guidance on CS [14] and the most recent ESC guidelines for the management of CS [15]. Traditionally regarded as a marker of anaerobic metabolism and tissue hypoxia, lactate is now recognized as a dynamic integrative signal reflecting the interaction between oxygen delivery, microcirculatory function, metabolic stress, adrenergic activation, and organ-specific clearance (Fig. 1) [[16], [17], [18], [19]]. The classic historical classification proposed by Cohen and Woods in the 1980s divided lactic acidosis into two major entities: type A, resulting from impaired tissue perfusion or oxygen delivery, and type B, characterized by lactate accumulation in the absence of overt hypoxia, often linked to metabolic derangements or impaired clearance [20]. Recently, building on this framework, Muller et al. introduced a mechanism-oriented classification that more precisely reflects the biological drivers of lactate elevation. This updated model delineates three dominant pathways [1]: increased pyruvate generation due to nonspecific acceleration of glycolysis, such as that induced by catecholamine excess, β-agonists, respiratory alkalosis, or metabolic stress [2]; reduced pyruvate utilization secondary to tissue hypoxia; and [3] impaired lactate clearance, most commonly observed in the setting of advanced hepatic or renal dysfunction (Table 1) [21]. However, in CS, these multiple mechanisms frequently coexist and overlap, substantially increasing the complexity of clinical interpretation.

**Table 1:** Mechanism leading to hyperlactatemia (inspired by Muller et al., 2023 [21] and [23]).

Mechanism of hyperlactatemia	Pathophysiological process	Examples / Typical causes	
Increased pyruvate production	Non-specific glycolysis stimulation	Catecholamines (mainly epinephrine), inhaled beta-agonists, respiratory alkalosis, pheochromocytoma	
	Malignancy-associated metabolic disturbances (*Warburg effect*)	Leukemia, lymphoma, or, less often, solid malignancies	
	Other causes influencing the redox potential of the cell	Alcoholism, diabetic ketoacidosis	
Decreased pyruvate utilization	Hypoxia-related hyperlactatemia (type A hyperlactatemia)	Crossing of an anaerobic threshold in skeletal muscle	Intensive muscle activity, generalized convulsions, hypothermic shivering
		Insufficient load of oxygen in blood	Hypoxemic hypoxia, profound anemia, CO intoxication
		Insufficient perfusion	Shock, regional ischemia (i.e., mesenteric ischemia), cardiac arrest, microvascular shunting
	Pyruvate-dehydrogenase dysfunction	Thiamine deficiency (beriberi), inherited enzymatic dysfunction	
	Mitochondrial dysfunction	Drugs (propofol, linezolid, metformin, etc.), inherited disorders, cyanide intoxication, sepsis	
Altered clearance of lactate	Liver failure, renal failure		

Beyond its prognostic value, lactate has gained increasing importance as a marker of treatment response and may even represent an adaptive metabolic substrate under conditions of cardiac stress [[22], [23], [24]]. This evolving understanding challenges the long-standing perception of lactate as a mere by-product of shock [25] and has important implications for clinical interpretation and therapeutic strategy [[26], [27], [28]].

## Pathophysiology of lactate in cardiogenic shock (Fig. 2)

### Lactate production under aerobic and anaerobic conditions

Lactate is continuously produced through the conversion of pyruvate by lactate dehydrogenase, even under aerobic conditions. Lactate production is primarily determined by glycolytic flux rather than oxygen availability per se [29,30]. Under resting conditions, the weight-adjusted daily production of lactate in humans is estimated to be 20 mmol/kg. Indeed, in a standard 70-kg adult, daily lactate production is approximately 1,400 mmol, originating from the skeletal muscles (25%), skin (25%), brain (20%), erythrocytes (20%), and gastrointestinal tract (10%) [25]. Thus, in physiological states, lactate serves as a central metabolic intermediate facilitating the transfer of carbon substrates and reducing equivalents between tissues, a concept formalized in the “lactate shuttle” theory [29,31]. In CS, hyperlactatemia reflects an imbalance between ongoing lactate production and its removal from the blood through excretion (e.g., urine, sweat) and metabolism (e.g., cellular uptake as a direct energy source, hepatic conversion to glucose), rather than a single mechanism. Impaired oxygen delivery, microcirculatory dysfunction, adrenergic stimulation, mitochondrial alterations, and reduced hepatic or renal clearance may all contribute simultaneously [18].

**Fig. 2:**
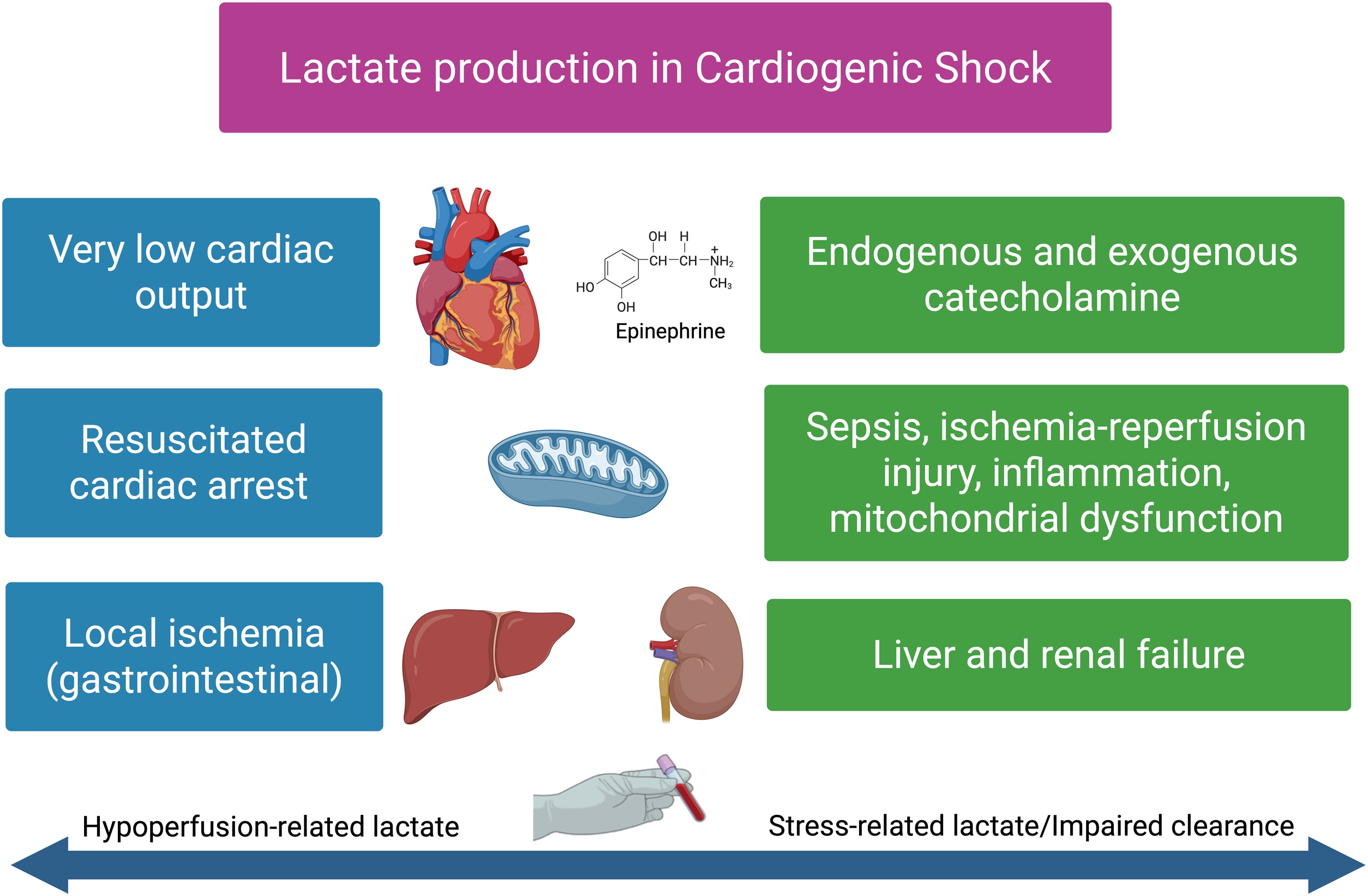
Mechanisms of lactate generation in cardiogenic shock (Adapted from [97]). In cardiogenic shock, lactate elevation results from both anaerobic and aerobic mechanisms. Profoundly reduced cardiac output, resuscitated cardiac arrest, and regional ischemia (particularly splanchnic) lead to anaerobic lactate production through tissue hypoxia. In parallel, endogenous and exogenous catecholamine stimulation promotes aerobic glycolysis. Post–cardiac arrest syndrome, ischemia–reperfusion injury, systemic inflammation, and mitochondrial dysfunction further contribute to lactate generation. Impaired hepatic and renal clearance exacerbate hyperlactatemia, resulting in persistent lactate elevation despite restored oxygen delivery.

### Global hypoperfusion and anaerobic glycolysis

The most intuitive mechanism of lactate elevation in CS is global hypoperfusion due to reduced cardiac output. When systemic oxygen delivery (DO₂) falls below a critical threshold, mitochondrial oxidative phosphorylation becomes insufficient to meet cellular energy demands, leading to a shift toward anaerobic glycolysis. Under these hypoxic conditions, glycolysis of glucose generates pyruvate, which is then preferentially converted into lactate (reduction by L-lactate dehydrogenase (LDH)), allowing regeneration of NAD^+^ and continuation of glycolysis [32]. This mechanism predominates in the early and most severe phases of CS and is typically associated with other markers of global hypoxia, such as low mixed venous oxygen saturation and metabolic acidosis [6]. Normal lactate/pyruvate (LP) ratio is around 10. An elevated lactate/pyruvate ratio suggests a decrease in cytoplasmic redox state toward increased NADH/NAD^+^, usually reflecting tissue hypoxia. In CS, Levy et al. found a value of 40+/-6 [33]. Consistent with this concept, Rimachi et al. demonstrated in CS patients that serial measurements of lactate and pyruvate revealed an increased lactate/pyruvate ratio during the early hypoperfusion phase, supporting the hypothesis that anaerobic metabolism is the primary driver of hyperlactatemia in the early stages of shock [34]. Moreover, Revelly et al., using infused ¹³C-labeled lactate (a stable, non-radioactive isotope tracer enabling quantification of lactate kinetics), demonstrated that in CS, hyperlactatemia is primarily driven by increased endogenous lactate production, whereas metabolic clearance appears comparatively less affected [35].

### Microcirculatory dysfunction and loss of hemodynamic coherence

Beyond macrocirculatory failure, and similarly to other types of shock, severe or persistent CS may induce profound microcirculatory alterations, including reduced functional capillary density, heterogeneous blood flow distribution, and impaired oxygen extraction [19]. These abnormalities may persist despite normalization of macrohemodynamic variables such as systemic blood pressure or cardiac output, leading to a loss of hemodynamic coherence between macro and microcirculation [36]. As a result, regional hypoxia and lactate production may occur even when global DO₂ appears adequate, explaining persistent hyperlactatemia despite apparent hemodynamic stabilization [37]. Indirect assessment of tissue perfusion can be performed using several bedside markers that correlate with lactate levels in early shock, such as urine output (though its interpretation is limited by the frequent use of diuretics for congestion management and the high prevalence of type 1 acute cardiorenal syndrome in CS), capillary refill time (CRT) [38], mottling score [39,40], and the venous-to-arterial CO₂ gap [41]. Interestingly, in a study by Merdji et al., CRT and skin mottling, two well-established clinical markers of microcirculatory dysfunction [40,42], were more strongly correlated with lactate levels than traditional macrocirculatory parameters, such as blood pressure or cardiac output in CS [43]. Similar to lactate, these readily available and cost-free bedside markers have demonstrated strong prognostic relevance in early CS [43,44], although their interpretation has recognized limitations [45].

### Adrenergic-driven aerobic glycolysis

CS is associated with intense endogenous catecholamine release and often requires administration of exogenous vasoactive agents. β-adrenergic stimulation increases glycolytic flux via activation of key enzymes and Na^+^/K^+^-ATPase activity, leading to increased lactate production even in the presence of sufficient oxygen availability [17,46]. This phenomenon, often referred to as aerobic glycolysis or stress hyperlactatemia, is particularly relevant in patients receiving epinephrine and may result in lactate elevation independent of tissue hypoxia, complicating clinical interpretation. Consistent with this mechanism, the OPTIMA-CC trial reported that epinephrine (a powerful β-adrenergic agonist) administration in acute myocardial infarction (AMI)–related CS was associated not only with higher heart rates and a greater risk of refractory CS, but also with substantially higher lactate concentrations, approximately twofold greater than those observed with norepinephrine or dobutamine, reinforcing the role of catecholamine-driven aerobic glycolysis [47]. In line with these findings, the study by Léopold et al. [48] also demonstrated potentially deleterious effects of epinephrine compared with norepinephrine in CS. Taken together, these data suggest that epinephrine should be avoided whenever possible in CS.

Conversely, in patients with acute-on-chronic heart failure–related CS receiving long-term non-selective β-blocker therapy, lactate levels at shock onset may be artificially attenuated, potentially leading to underestimation of shock severity when relying on lactate alone. Supporting this observation, prior studies have shown that patients on chronic or premorbid β-blocker therapy are less likely to develop marked hyperlactatemia (≥4 mmol/L) during sepsis or septic shock and exhibit lower initial lactate concentrations compared with patients not receiving β-blockers [49,50]. A similar pattern is observed in β-blocker poisoning, in which early lactate elevation often seems disproportionately low relative to the clinical severity of CS [51]. This effect likely reflects attenuation of β₂-adrenergic–mediated aerobic glycolysis and reduced lactate generation [17]. However, although significantly lower lactate levels have been reported in some studies among patients with acute-on-chronic heart failure (HF)-CS compared with those with de-novo HF–CS [17], this difference is not consistently observed across studies [52,53]. One potential explanation is that most patients with chronic heart failure are currently treated with cardioselective β-blockers rather than non-selective agents, which may differentially influence lactate production [54].

### Impaired lactate clearance

Lactate clearance is primarily mediated by the liver (60%) via gluconeogenesis and oxidation, with additional contributions from the kidneys (30%) through similar mechanisms, with urinary excretion serving only a minor role in clearance (<10%) [54] and other organs. In an ancillary analysis of the CardShock study, patients with abnormal liver function tests consistent with hypoxic hepatitis (defined by an ALT increase >20%) exhibited significantly higher lactate levels in CS, which remained elevated throughout the first 24 h compared with patients without hypoxic hepatitis [55]. Hepatic lactate uptake is highly flow-dependent and requires adequate oxygenation. In CS, hepatic hypoperfusion, venous congestion, ischemic hepatitis, and acute kidney injury frequently impair lactate clearance, contributing to persistent hyperlactatemia even after partial restoration of perfusion [[56], [57], [58], [59]].

Interestingly, existing evidence indicates that hemofiltration during continuous renal replacement therapy (CRRT) has only a negligible impact on lactate clearance [60,61].

### Lactate as a key oxidative fuel for the heart during systemic stress

The heart has an exceptionally high and continuous energy demand, requiring sustained production of adenosine triphosphate (ATP) to preserve contractile function, with an estimated consumption of approximately 1 mmol of ATP per second. In the absence of ongoing ATP resynthesis, myocardial ATP stores would be exhausted within 2–10 seconds, resulting in rapid contractile failure. Continuous ATP generation is therefore indispensable for maintaining normal cardiac performance [62]. Myocardial energy production relies almost exclusively on mitochondrial oxidative phosphorylation, which depends on oxygen utilization within mitochondria and accounts for more than 90% of total ATP generation. This reliance underlies the remarkably high myocardial oxygen extraction, such that venous blood draining from the coronary sinus exhibits an oxygen saturation of ≈40%, representing the most deoxygenated venous blood in the body [63]. This substantial energetic demand is met through metabolic flexibility, allowing the myocardium (a “metabolic omnivore”) to oxidize a range of substrates including fatty acids, glucose, ketone bodies, and lactate to sustain ATP production via mitochondrial oxidative phosphorylation (re 2 and 3) [64]. Once generated, approximately 60%–70% of myocardial ATP is devoted to mechanical contraction, while the remaining 30%–40% supports ionic homeostasis, predominantly through ATP-dependent ion transport processes, particularly sarcoplasmic reticulum Ca²^+^-ATPase–mediated calcium handling [65].

In this context, lactate should not be regarded solely as a marker of metabolic failure. Experimental and physiological studies demonstrate that lactate is a preferred oxidative substrate for the stressed myocardium and can support ATP generation when fatty acid and glucose metabolism are impaired [[66], [67], [68]].

In a healthy porcine model, sodium lactate infusion has been reported to increase cardiac output, accompanied by a concurrent rise in whole-body oxygen consumption, consistent with oxidative metabolism of lactate [69]. In a porcine model of ischemic CS, lactate infusion has been shown to improve cardiac output, stroke volume, and myocardial mechanical efficiency, while enhancing mitochondrial respiration, particularly when combined with inotropic stimulation [22,70]. In a rodent model of septic shock, Levy et al. demonstrated that systemic lactate deprivation adversely affected myocardial energetics, cardiovascular performance, and survival, underscoring lactate’s role as a critical metabolic substrate [71]. Consistent findings have also been reported in rodent models of hemorrhagic shock, in which lactate supplementation improved hemodynamic and metabolic outcomes [72].

In humans, lactate has similarly demonstrated cardiometabolic benefits [26]. In healthy volunteers, infusion of half-molar sodium lactate enhanced cardiac performance, as evidenced by increases in cardiac output, stroke volume, and left ventricular ejection fraction, compared with sodium-matched hypertonic saline, suggesting a direct energetic and functional advantage beyond osmotic or volume effects [73]. In patients with acute heart failure, infusion of half-molar sodium lactate has been shown to increase cardiac output and tricuspid annular plane systolic excursion (TAPSE) compared with control treatment [74]. Similar benefits have been reported in patients after coronary artery bypass grafting, in whom lactate infusion significantly increased cardiac index relative to control [75].

**Fig. 3:**
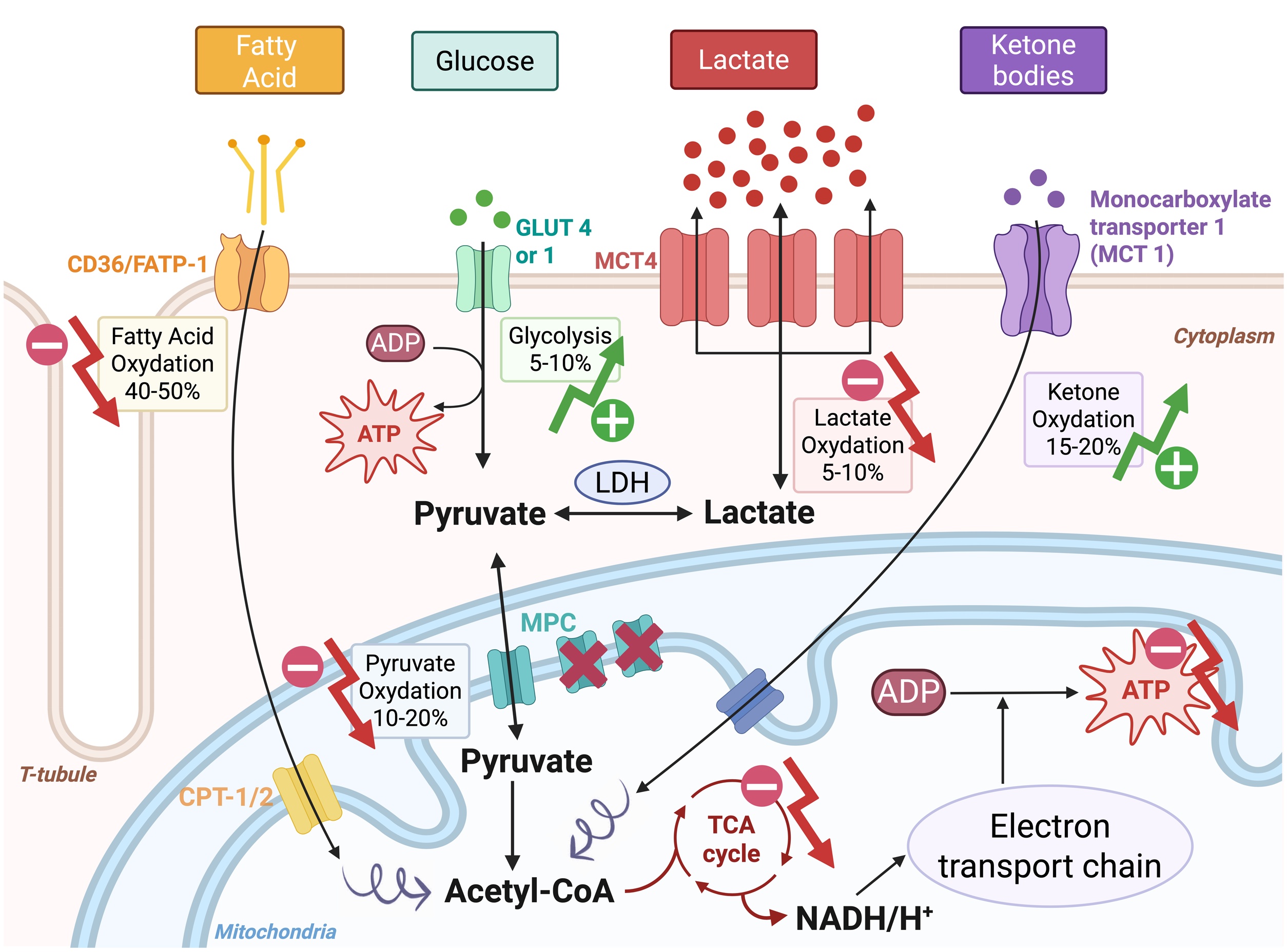
Overview of energy metabolism during Cardiogenic shock (Adapted from [64] and [11]). In cardiogenic shock, profound alterations in myocardial substrate utilization and mitochondrial metabolism occur. Fatty acid oxidation is reduced, while glycolytic flux is increased, leading to elevated cytosolic pyruvate levels. Concomitantly, mitochondrial pyruvate carrier (MPC) activity is downregulated, limiting pyruvate entry into mitochondria and diverting its metabolism toward lactate production via LDH, a bidirectional enzyme regulated by intracellular pyruvate and lactate concentrations. In parallel, the expression and activity of MCT4, a monocarboxylate transporter primarily involved in lactate export, are upregulated, contributing to disturbed pyruvate–lactate homeostasis and elevated circulating lactate levels. Additional remodeling includes changes in ketone oxidation, amino acid oxidation, and glucose oxidation, reflecting an adaptive but often insufficient attempt to preserve ATP production under conditions of impaired oxygen delivery and mitochondrial dysfunction. Arrows indicate the direction of change in pathway activity (upward, increased; downward, decreased), and numbers represent the approximate relative contribution of each metabolic pathway to overall myocardial ATP production in cardiogenic shock

These data suggest that lactate can serve as a preferential oxidative substrate under stress conditions. However, whether systemic hyperlactatemia in CS represents a protective adaptive response or merely reflects metabolic stress occurring in parallel with substrate utilization remains uncertain. The presence of lactate-consuming organs does not imply that its systemic elevation is intrinsically beneficial (Fig. 3). Therefore, although experimental and preliminary clinical data suggest potential metabolic benefits of lactate infusion, evidence remains insufficient to support routine therapeutic use in CS.

## Prognostic Value of Lactate in Cardiogenic Shock

### Baseline lactate

Admission lactate has long been associated with mortality in CS. Thresholds around 3.0–3.5 mmol/L consistently identify patients at high risk of short-term death [[76], [77], [78]]. In one of the largest studies evaluating the prognostic value of lactate levels in AMI-CS, the optimal baseline lactate cutoff for predicting 30-day mortality was 5.0 mmol/L [7]. However, baseline lactate is influenced by pre-hospital delay, early resuscitation, and catecholamine exposure, limiting its ability to discriminate reversibility once treatment has begun.

It should be noted that arterial and central venous lactate concentrations are generally closely correlated in shock states; however, regional gradients may exist in the presence of severe splanchnic ischemia or impaired hepatic clearance. In clinical practice, serial measurements from the same sampling site are preferable to avoid misinterpretation of trends. In cardiac intensive care patients with a high inotrope/vasopressor score, Vanderbriele et al. founded a close correlation between radial and mixed venous lactate (from pulmonary artery catheter) [79]. Nevertheless, it should be emphasized that these findings cannot be extrapolated to peripheral venous lactate, which may be influenced by local tissue metabolism, perfusion gradients, and regional metabolic activity. Indeed, several studies have reported that the difference between peripheral venous and arterial lactate measurements can be substantial, with venous values ranging from more than 3.5 mmol/L higher to 2.3 mmol/L lower than arterial levels. Such wide variability highlights the potential for clinically misleading interpretations when these sampling sites are used interchangeably [80].

### Dynamic lactate monitoring

Serial lactate measurements provide superior prognostic information compared with isolated values [7,[81], [82], [83]]. Persistent hyperlactatemia or rising lactate trajectories identify patients with refractory shock, ongoing microcirculatory dysfunction, or impaired metabolic recovery, even when macrocirculatory targets appear achieved. In a substudy of the IABP-SHOCK II trial and its associated registry, investigators evaluated the prognostic value of arterial lactate at baseline, 8-h lactate, and early lactate clearance for predicting 30-day mortality in patients with AMI–related CS. Lactate measured at 8 h demonstrated superior prognostic accuracy compared with both baseline lactate and lactate clearance. Among all lactate-derived metrics, an 8-h lactate threshold of 3.1 mmol/L after initial management provided the best discriminatory performance for early risk stratification in CS [7].

### Lactate reduction and lactate load

A reduction in lactate levels during the first 12–24 h has consistently been associated with improved survival in CS. Lactate appears to provide its strongest prognostic signal during the early phase of CS [9], with its predictive accuracy declining beyond the first 24 h [84]. However, focusing solely on the rate of lactate decline may underestimate the prognostic burden of sustained hyperlactatemia. To address this limitation, the concept of “normalized lactate load”, which integrates both the magnitude and duration of lactate elevation, has demonstrated superior prognostic performance in critically ill patients and in CS cohorts [85,86].

In a post hoc analysis of the DOREMI trial (Dobutamine Compared to Milrinone in the Treatment of Cardiogenic Shock), complete lactate clearance emerged as the strongest predictor of survival across all evaluated time points. The prognostic impact increased over time, with odds ratios for survival increasing from ≈2.5 at 8 h to ≈5.5 at 24 h [81].

### Lactate in risk stratification scores

Lactate has been incorporated into most validated CS risk scores. In the CardShock score, lactate ≥2 mmol/L was independently associated with 30-day mortality [3]. In the CLIP (cystatin C, lactate, IL-6, and NT-proBNP) score, lactate carries one of the highest normalized coefficients, indicating a major contribution to 30-day mortality prediction. Notably, during the development of this score, lactate was identified as one of the four most robust predictors from an initial pool of 58 candidate biomarkers [87]. Similarly, in the Cardiogenic Shock Score, lactate ≥4 mmol/L identifies patients at markedly higher risk of short-term mortality [88]. In patients receiving VA-ECMO, both the ENCOURAGE and SAVE scores include lactate as a major determinant of outcome [89,90]. Across all models, lactate consistently remains one of the strongest predictors of mortality.

**Fig. 4:**
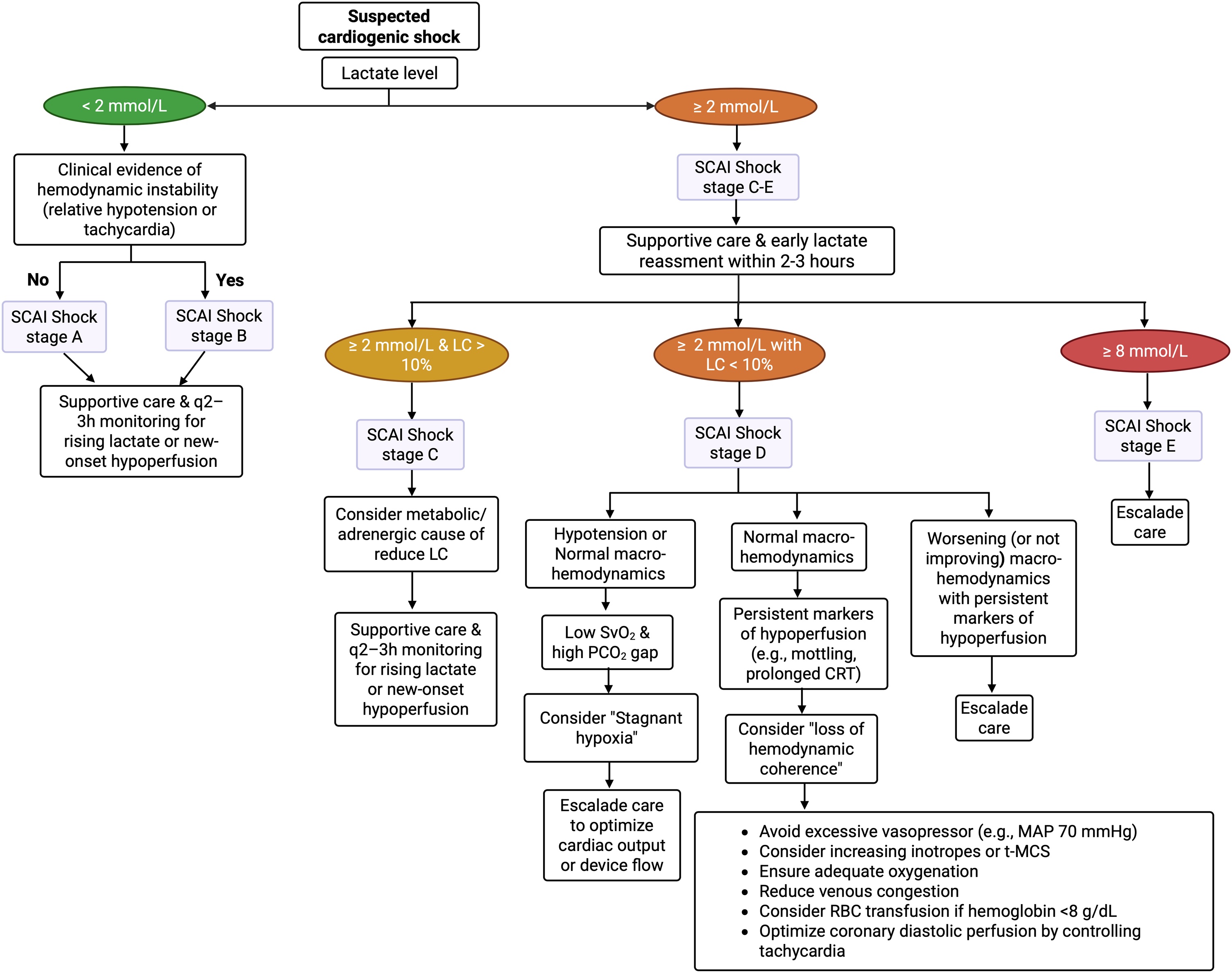
Bedside diagnostic algorithm for the integrated assessment of lactate kinetics and peripheral perfusion markers in cardiogenic shock (Inspired from [28]). CRT: capillary refill time; LC: lactate clearance; MAP: mean arterial pressure; t-MCS: temporary mechanical circulatory support; RBC: red blood cell.

Based on these data, and drawing on the framework proposed by Naidu et al., we propose a bedside diagnostic algorithm that integrates lactate kinetics with peripheral perfusion markers in CS (Fig. 4) [28].

### Lactate and mechanical circulatory support

CS patients supported with venoarterial extracorporeal membrane oxygenation (VA-ECMO) present unique challenges. They commonly experience substantial ischemic-reperfusion injuries, receive high doses of catecholamines, and frequently suffer from both liver and kidney dysfunction based on the severity and duration of low cardiac output syndrome. Additionally, approximately 50% of these patients have undergone cardiac arrest [91]. Furthermore, the degree and reversibility of myocardial failure and complications associated with VA-ECMO may influence the relationship between lactate levels, changes in lactate, and time to lactate normalization, and early or late survival. Pre-VA-ECMO lactate levels have been linked to prognosis [92,93] or included in several prognostic scores [89,94]. A recent meta-analysis and systematic review have brought to light that a reduction in lactate levels is associated with improved prognoses in CS, which includes a subgroup of patients supported by VA-ECMO [8]. In a post hoc analysis of the HYPO-ECMO study, Levy et al. found [95] a consistent and significant association between lactate levels, whether assessed at baseline or during ICU treatment, and the risk of mortality, which underscores the pivotal prognostic relevance of lactate levels in patients with CS undergoing VA-ECMO therapy. Beyond absolute lactate values, the study provided novel insights into lactate kinetics, demonstrating that the occurrence of a secondary lactate peak within the first 7 days after VA-ECMO initiation (observed in approximately 24% of patients) was independently associated with worse outcomes, regardless of baseline lactate levels.

In a DanGer-SHOCK substudy, patients randomized to microaxial flow pump support exhibited faster lactate clearance and achieved complete normalization approximately 12 h earlier than those receiving standard care, despite comparable baseline lactate levels [96].

## Conclusion

Lactate is a central integrative biomarker in CS, reflecting hypoperfusion, metabolic stress, adrenergic activation, and impaired clearance. Dynamic assessment of lactate kinetics rather than static values alone provides robust prognostic information and insight into shock reversibility. In the era of t-MCS, lactate remains both a guide and a potential therapeutic target, underscoring its pivotal role in the management of cardiogenic shock.

## Authors’ contributions

BL: Conceptualization, Methodology, Supervision, Validation, Visualization, Writing original draft.

GH: Validation, Writing – original draft, Writing – review & editing.

HM: Writing – original draft, Writing – review & editing.

## Consent for publication

Not applicable.

## Ethics approval and consent to participate

Not applicable.

## Funding

Not applicable.

## Availability of data and material

Not applicable.

## Declaration of competing interest

Levy B reports was provided by Nancy Regional University Hospital Center. Levy reports a relationship with AOP Orphan Pharmaceuticals GmbH that includes: board membership, consulting or advisory, funding grants, speaking and lecture fees, and travel reimbursement. Levy reports a relationship with Viatris that includes: board membership, consulting or advisory, speaking and lecture fees, and trave reimbursement. None If there are other authors, they declare that they have no known competing financial interests or personal relationships that could have appeared to influence the work reported in this paper.
